# Cross-sectional validation of the PROMIS-Preference scoring system

**DOI:** 10.1371/journal.pone.0201093

**Published:** 2018-07-31

**Authors:** Janel Hanmer, Barry Dewitt, Lan Yu, Joel Tsevat, Mark Roberts, Dennis Revicki, Paul A. Pilkonis, Rachel Hess, Ron D. Hays, Baruch Fischhoff, David Feeny, David Condon, David Cella

**Affiliations:** 1 Department of General Internal Medicine, University of Pittsburgh Medical Center, Pittsburgh, Pennsylvania, United States of America; 2 Department of Engineering and Public Policy and Institute for Politics and Strategy, Carnegie Mellon University, Pittsburgh, Pennsylvania, United States of America; 3 Department of Medicine, Long School of Medicine, University of Texas Health Science Center at San Antonio, San Antonio, Texas and Department of Population Health, Dell Medical School, University of Texas at Austin, Austin, Texas, United States of America; 4 Department of Health Policy and Management, University of Pittsburgh, Pittsburgh, Pennsylvania, United States of America; 5 Outcomes Research, Evidera, Bethesda, Maryland, United States of America; 6 Department of Psychiatry, University of Pittsburgh School of Medicine, Pittsburgh, Pennsylvania, United States of America; 7 Division of Health System Innovation and Research, University of Utah Schools of the Health Sciences, Salt Lake City, Utah, United States of America; 8 Division of General Internal Medicine & Health Services Research, UCLA, Los Angeles, California, United States of America; 9 Department of Economics, McMaster University, Hamilton, Ontario, Canada; 10 Department of Medical Social Sciences, Northwestern University Feinberg School of Medicine, Chicago, Illinois, United States of America; National University of Singapore, SINGAPORE

## Abstract

**Objectives:**

The PROMIS-Preference (PROPr) score is a recently developed summary score for the Patient-Reported Outcomes Measurement Information System (PROMIS). PROPr is a preference-based scoring system for seven PROMIS domains created using multiplicative multi-attribute utility theory. It serves as a generic, societal, preference-based summary scoring system of health-related quality of life. This manuscript evaluates construct validity of PROPr in two large samples from the US general population.

**Methods:**

We utilized 2 online panel surveys, the PROPr Estimation Survey and the Profiles-Health Utilities Index (HUI) Survey. Both included the PROPr measure, patient demographic information, self-reported chronic conditions, and other preference-based summary scores: the EuroQol-5D (EQ-5D-5L) and HUI in the PROPr Estimation Survey and the HUI in the Profiles-HUI Survey. The HUI was scored as both the Mark 2 and the Mark 3. Known-groups validity was evaluated using age- and gender-stratified mean scores and health condition impact estimates. Condition impact estimates were created using ordinary least squares regression in which a summary score was regressed on age, gender, and a single health condition. The coefficient for the health condition is the estimated effect on the preference score of having a condition vs. not having it. Convergent validity was evaluated using Pearson correlations between PROPr and other summary scores.

**Results:**

The sample consisted of 983 respondents from the PROPr Estimation Survey and 3,000 from the Profiles-HUI survey. Age- and gender-stratified mean PROPr scores were lower than EQ-5D and HUI scores, with fewer subjects having scores corresponding to perfect health on the PROPr. In the PROPr Estimation survey, all 11 condition impact estimates were statistically significant using PROPr, 8 were statistically significant by the EQ-5D, 7 were statistically significant by HUI Mark 2, and 9 were statistically significant by HUI Mark 3. In the Profiles-HUI survey, all 21 condition impact estimates were statistically significant using summary scores from all three scoring systems. In these samples, the correlations between PROPr and the other summary measures ranged from 0.67 to 0.70.

**Conclusions:**

These results provide evidence of construct validity for PROPr using samples from the US general population.

## Introduction

Patient reports about functioning and well-being, or health-related quality of life (HRQoL), are important outcomes of health care and policy [1,2]. Measures of HRQoL can be disease-targeted, providing detailed measurement about symptoms, treatment effects, and side effects relevant to a particular condition. Measures can also be generic, providing an overall description of health not limited to one organ system or disease [3]. The proliferation of HRQoL measures, however, has made it difficult to compare results across studies that use different measures [4,5].

HRQoL can be categorized further using health profile measures or preference-based measures. Profile measures provide a description of multiple domains of health such as physical functioning, mental health, and pain. These measures provide multiple scores–one for each domain of health. Preference-based measures also cover multiple health domains but are combined into a single summary preference-based score, on a scale where 0 is “dead” and 1 is “full health”) scale. Preference-based scoring allows for estimates of quality-adjusted life years (QALYs) that afford comparisons among treatment options in clinical decision-making and economic analysis [2, 6–8].

The most widely used generic preference-based measures include the EuroQol-5D (EQ-5D) [9], Health Utilities Index (HUI) [10–11], SF-6D [12], and the Quality of Well-being Index [13]. Each, however, has some limitations: (1) large proportions of the respondents scoring at the very top or very bottom of the scale (i.e., ceiling effects in the very healthy or floor effects in the very ill), (2) imprecise measurement for individuals, (3) poorly-worded questions such as those that combine concepts (double-barreled items), and (4) lack of coverage of the full range of health [14]. These limitations arise from the descriptions of health used in these measures and not the method of scoring.

Recent efforts to develop the next generation of health profile measures using Item Response Theory (IRT) [15] have created an opportunity to leverage these improvements into preference-based measures [14]. Most notably, IRT has been used to develop the Patient-Reported Outcomes Measurement Information System (PROMIS) with support from the National Institutes of Health [16,17]. PROMIS addresses several limitations of the existing generic preference measures including: (1) capturing a wider range of each health domain, (2) measuring individual health status with greater precision, and (3) using rigorously designed and tested questions. A preference-based scoring system for PROMIS would create the possibility of simultaneously collecting both health profile and preference-based scores.

Using input from measurement experts and community members, we developed a preference scoring system based on 7 domains from the PROMIS measure, known as PROMIS-Preference (PROPr) [18, 19]. The scoring algorithm was estimated using a large representative sample of the US non-institutionalized population [20]. PROPr is the first preference-based summary score to link directly preference-based functions to health domains as measured by IRT. As such, PROPr gains many of the advantages of an IRT-based descriptive system, including flexible administration of items from the health domains used to construct PROPr.

Before any measure can be adopted for widespread use, its validity needs to be demonstrated [3]. We used 2 cross-sectional surveys of the general US population to evaluate the construct validity of the PROPr.

## Methods

### Data sources

The first dataset was the PROPr Estimation Survey collected online by ICF and SurveyNow, which maintain a panel of pre-validated individuals, primarily for market research. Data were collected in the spring of 2016. The survey sample was intended to be representative of the US population by age, gender, race, ethnicity, education, and income consistent with the 2010 census. As compensation for completing the survey, participants could choose from several rewards, including gift cards and reward program points. The survey was approved by the Institutional Review Board at ICF International (Study #FWA00002349). Responses were de-identified before the authors received them. A full description of the survey is available in the PROPr technical report [21]. Data used in these analyses are available online at the Open Science Framework [22].

The second dataset was the Profiles-HUI survey, collected online using the Op4G internet panel. Op4G maintains a US national sample and participants are required to update demographic information regularly. The sample was ascertained using quotas for region, race/ethnicity, education, and age-gender strata consistent with the 2010 census. Study participants received nominal incentives from Op4G for completing the survey. The specific nature and value of the incentive varied but did not exceed $10. The survey was approved by the Institutional Review Board at Northwestern University (Study #STU00016635). Data used in these analyses are available online at in Dataverse [23].

This study used de-identified data provided by the survey companies. Each study was analyzed separately. Participants in the PROPr Estimation Survey were randomized to receive the EQ-5D-5L, HUI, health conditions, or PROMIS Global questionnaire first. All participants then received the PROMIS-29 and PROMIS Cognitive Function short form (which provided the data on the 7 domains necessary for PROPr) and completed the PROPr valuation exercises. After the valuation exercises, the participant completed the other sections (EQ-5D-5L, HUI, health conditions, and PROMIS Global questionnaire) in random order. All participants in the Profiles-HUI survey completed the same survey form. They first completed a set of socio-demographic and comorbidity items, followed by the PROMIS Global form, a selection of PROMIS items from various banks, and then the HUI. Both surveys asked respondents for demographic information including their age and gender, and about their health conditions by using standardized language from the National Health Interview Survey (e.g., “Have you EVER been told by a doctor or other health professional that you had coronary heart disease?”) [24]. The PROPr Estimation Survey included 11 health conditions and the Profiles-HUI survey included 21 health conditions.

### Measures

#### PROPr

PROPr is based on levels of functioning for 7 PROMIS domains: Cognitive Function, Depression, Fatigue, Pain Interference, Physical Function, Sleep Disturbance, and Ability to Participate in Social Roles and Activities. The PROMIS questions refer to the respondent’s own health “in the last 7 days” and have 5 response options. The PROPr Estimation survey used the standardized 4-item short forms for each of these domains. The Profiles-HUI survey collected 8 to 13 items per domain. Domains were scored by the scoring service on Assessment Center incorporating the default IRT parameters for each item [25]. The PROPr scoring algorithm was developed from standard gamble valuations from a US sample [20]. Possible PROPr scores range from -0.022 to 1.0.

#### EQ-5D-5L

The EQ-5D-5L was collected in the PROPr Estimation survey. The EQ-5D-5L questions refer to “your health today.” The EQ-5D descriptive system has 5 domains (mobility, self-care, usual activities, pain/discomfort, and anxiety/depression). The PROPr Estimation Survey used the EQ-5D-5L version each with 5 response options [9, 26]. For this study, we applied the EQ-5D-3L crosswalk link function to the US time trade-off value set [27]. Possible scores range from -0.109 to 1.0.

#### Health Utilities Index (HUI)

The self-administered HUI questionnaire that allows scoring of both Mark 2 and Mark 3 was included in both surveys [10,11]. HUI questions refer to “your level of ability or disability during the past week.” The HUI Mark 2 defines health status on 6 attributes (sensation, mobility, emotion, cognition, self-care, and pain). The HUI Mark 3 defines health on 8 attributes (vision, hearing, speech, ambulation, dexterity, emotion, cognition, and pain). Scoring algorithms for both HUI Mark 2 and HUI Mark 3 were derived from standard gamble assessments made by adults in community samples in Hamilton, Ontario, and employ multiplicative multi-attribute utility functions. HUI Mark 2 scores range from -0.03 to 1.0; HUI Mark 3 scores range from -0.36 to 1.0.

### Statistical analysis

Each survey was analyzed separately. We calculated percentages for categorical demographic and health condition variables. We also generated histograms of summary scores for each sample. Convergent validity was evaluated using Pearson correlations between summary scores within-subjects (i.e., comparing the PROPr, HUI, and EQ-5D scores for each participant, in each of the two datasets).

Known-groups validity was evaluated using age- and gender-stratified mean scores for all summary scores. We expected PROPr to show the same patterns in age- and gender-stratified mean scores as other summary scores. Known-groups validity was also evaluated using health condition impact estimates that were created using ordinary least squares regression in which a summary score was regressed on age, gender, and a single health condition. The coefficient for the health condition is the estimated effect on the preference score of having a condition vs. not having it. A separate analysis was done for each condition.

All analyses were performed using SAS 9.4 (The SAS Institute, Cary, NC).

## Results

Table 1 summarizes the demographic characteristics of each sample. There were 983 respondents in the PROPr Estimation survey sample and 3000 respondents in the Profiles-HUI survey sample. For comparison, we display age, gender, and race/ethnicity information from the 2010 US census [28] and self-reported chronic condition prevalence from the 2016 National Health Interview Survey [29].

**Table 1:** Demographic information from the PROPr Estimation and Profiles-HUI surveys.

**Gender (%)**	**PROPr Estimation Survey**	**Profiles-HUI Survey**	**2010 US Census**
Female	54	51	51
Race (%)			
White	77	71	64
Black	12	17	12
Asian	4	10	5
Ethnicity (%)			
Hispanic	16	17	16
Education (%)			
< High School	12	14	11
High School Grad or Equivalent	25	31	30
> High School	63	55	59
Age (%)			
18–24 Years	11	13	12
25–34 Years	17	18	17
35–44 Years	10	18	18
45–54 Years	17	19	20
55–64 Years	19	16	16
65–74 Years	13	9	10
75–84 Years	7	6	6
85+ Years	6	2	3
Chronic Health Conditions (%)			National Health Interview Survey 2016
Coronary Heart Disease	2	Not asked	5
Angina (Angina Pectoris)	1	Not asked	2
Heart Attack (Myocardial Infarction)	1	5	4
Chest Pain (Angina)	Not asked	10	Not asked
Hardening of the Arteries (Coronary Artery Disease)	Not asked	4	Not asked
Heart Failure or Congestive Heart Failure	Not asked	4	Not asked
Stroke	6	Not asked	4
Stroke or Transient Ischemic Attack (TIA)	Not asked	3	Not asked
Emphysema	1	Not asked	2
Chronic Obstructive Pulmonary Disease (COPD)	4	5	4
Asthma	14	17	14
Cancer or Malignancy of any Kind	16	5	11
Arthritis/Gout/Lupus/Fibromyalgia	26	Not asked	28
Arthritis or Rheumatism	Not asked	20	Not asked
Seizure Disorder or Epilepsy	6	Not asked	Not asked
Diabetes or Sugar Diabetes	19	11	11
High Blood Pressure (Hypertension)	Not asked	34	35
Liver Disease/Hepatitis/Cirrhosis	Not asked	4	Not asked
Kidney Disease	Not asked	3	Not asked
Migraines or Severe Headaches	Not asked	16	Not asked
Depression	Not asked	24	Not asked
Anxiety	Not asked	21	Not asked
Alcohol or Drug Problem	Not asked	5	Not asked
Sleep Disorder	Not asked	13	Not asked
HIV or AIDS	Not asked	1	Not asked
Spinal Cord Injury	Not asked	3	Not asked
Multiple Sclerosis	Not asked	2	Not asked

**Fig 1:**
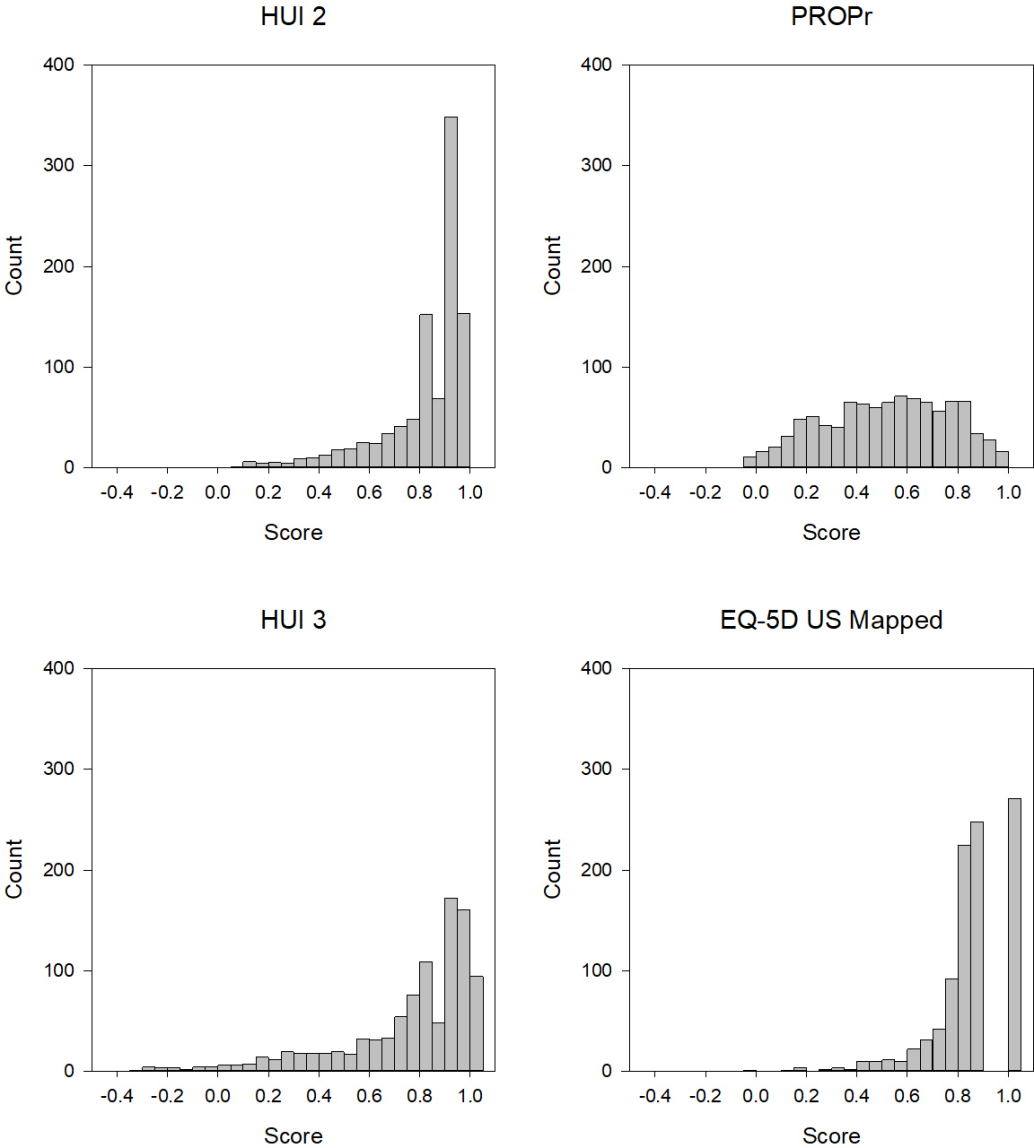
Distribution of summary scores in the PROPr Estimation sample. HUI 2 is the Health Utilities Index Mark 2, HUI 3 is the Health Utilities Index Mark 3, EQ-5D US Mapped is the Euroqol-5D-5L mapped to the US valuation set, PROPr is the PROMIS-Preference score.

**Fig 2:**
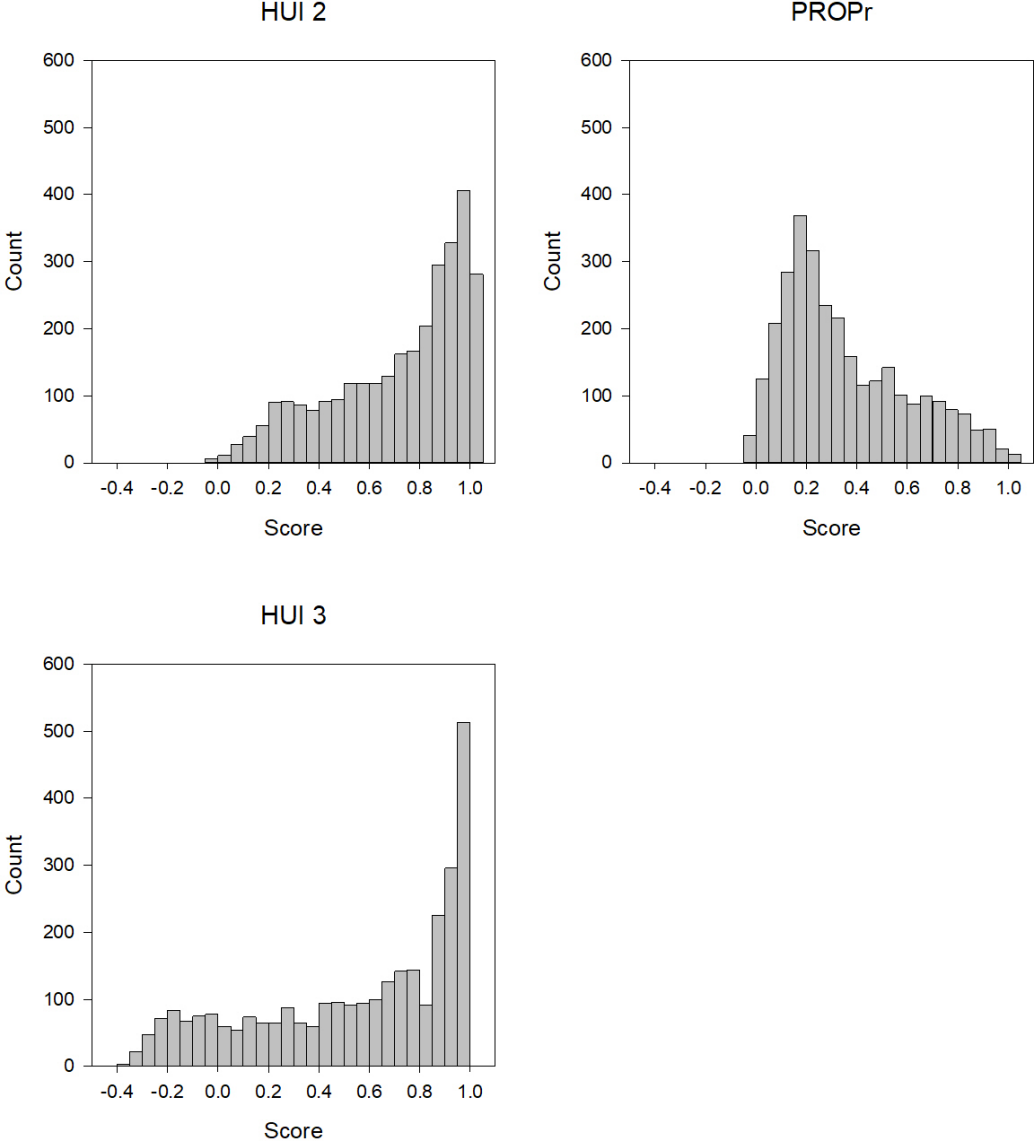
Distribution of summary scores in the Profiles-HUI sample. HUI 2 is the Health Utilities Index Mark 2, HUI 3 is the Health Utilities Index Mark 3, PROPr is the PROMIS-Preference score.

Figs 1 and 2 illustrate the distribution of summary scores in the PROPr Estimation and Profiles-HUI samples, respectively. The EQ-5D, HUI Mark 2, and HUI Mark 3 all have ceiling effects in these samples. The percent of respondents in the PROPr Estimation survey with a score at the ceiling was 28% for the EQ-5D, 11% for the HUI Mark 2, 10% for the HUI Mark 3, and 2% for PROPr. The percent of respondents in the Profiles-HUI survey with a score at the ceiling was 9% for the HUI Mark 2, 8% for the HUI Mark 3, and 0.4% for PROPr.

Tables 2 and 3 summarize the Pearson correlations between summary scores. In these samples, the correlation between PROPr and the other summary measures ranged from 0.66 to 0.70. The correlations between HUI Mark 2 and HUI Mark 3 are inflated because some of the same questions were used to create both scores.

**Table 2:** Pearson correlations between summary scores in the PROPr Estimation survey.

	PROPr	HUI Mark 3	HUI Mark 2
**EQ-5D**	0.70	0.79	0.72
**HUI Mark 2**	0.66	0.91	
**HUI Mark 3**	0.67		

**Table 3:** Pearson correlations between summary scores in the Profiles-HUI survey.

	PROPr	HUI Mark 3
**HUI Mark 2**	0.67	0.93
**HUI Mark 3**	0.70	

**Fig 3:**
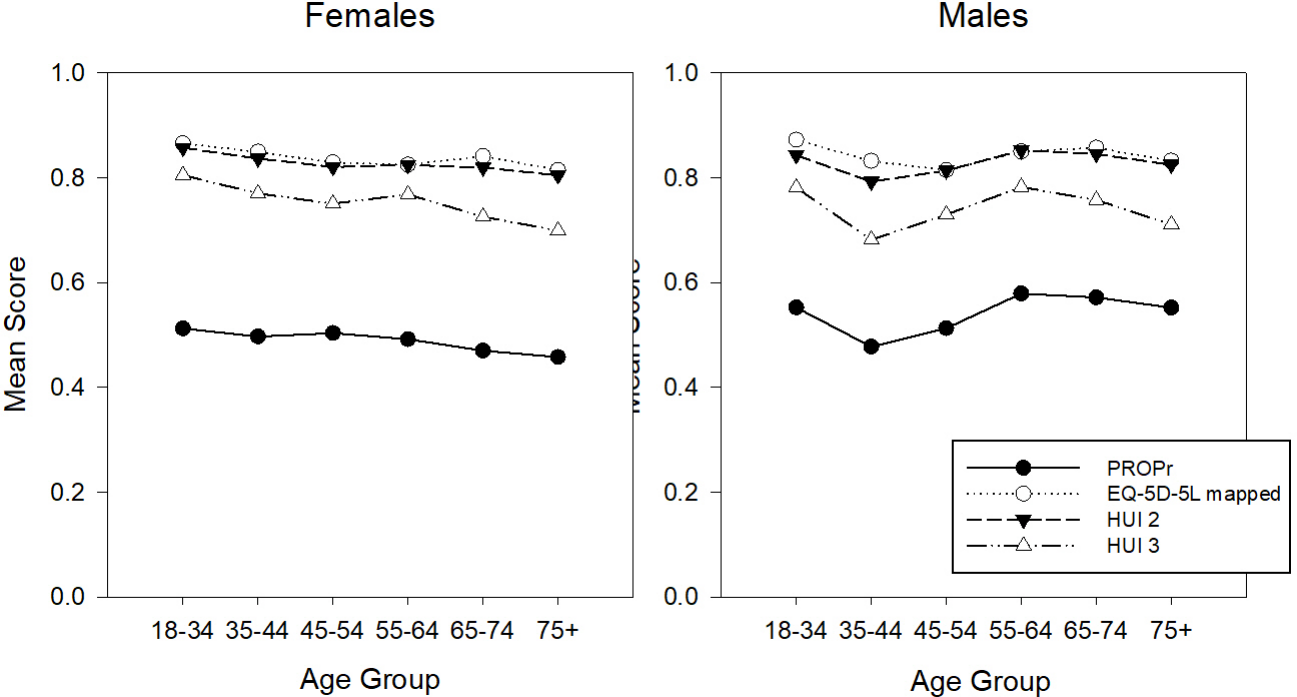
Age- and gender-stratified mean scores in the PROPr Estimation sample. HUI 2 is the Health Utilities Index Mark 2, HUI 3 is the Health Utilities Index Mark 3, EQ-5D US Mapped is the Euroqol-5D-5L mapped to the US valuation set, PROPr is the PROMIS-Preference score.

**Fig 4:**
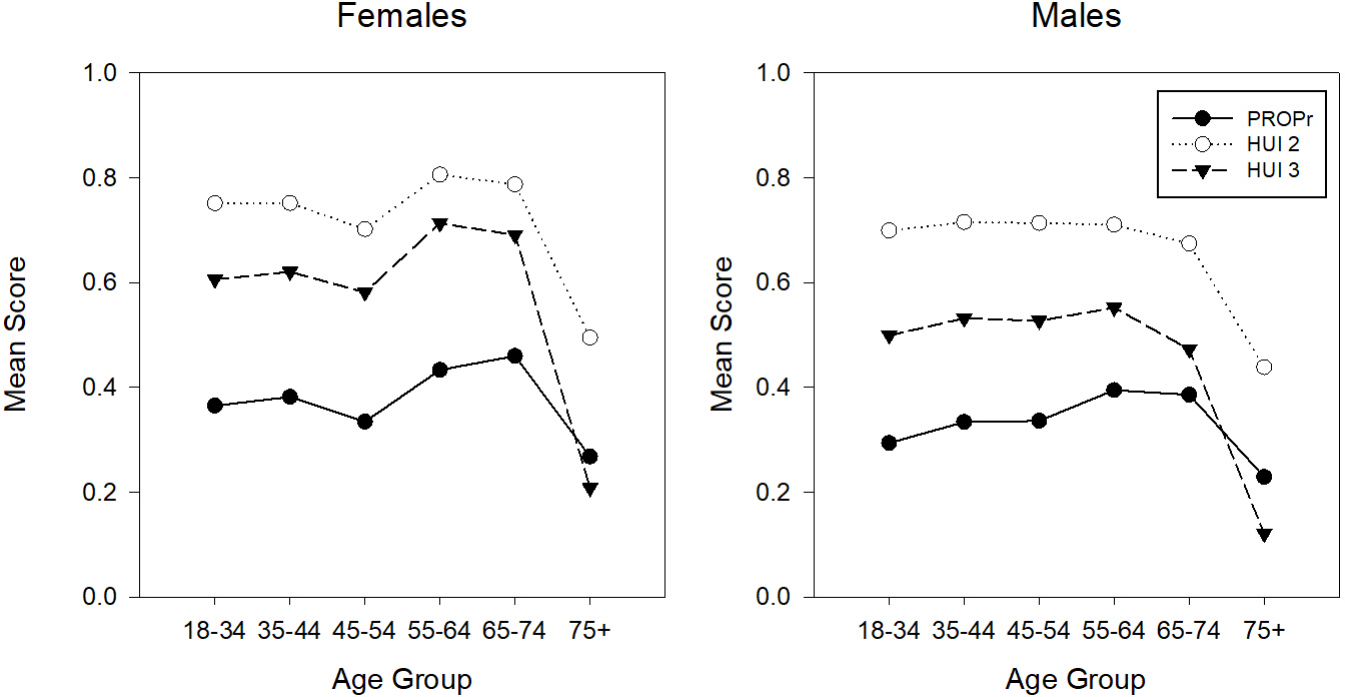
Age- and gender-stratified mean scores in the Profiles-HUI sample. HUI 2 is the Health Utilities Index Mark 2, HUI 3 is the Health Utilities Index Mark 3, PROPr is the PROMIS-Preference score.

PROPr, which does not have a ceiling effect in these samples, has the lowest age- and gender-stratified mean scores. Figs 3 and 4 illustrate the age- and gender- adjusted mean scores in the PROPr Estimation and Profiles-HUI samples, respectively. In nationally representative samples, health utility scores are usually lower in females than males and decrease with increasing age [30–32]. In the PROPr Estimation sample, males have lower scores than females in the younger age groups whereas females have lower scores in the older age groups. The association between age group and mean score is not monotonic in this sample though all summary scores generally change in the same direction between groups; e.g., all summary scores for 45–54 year old males are lower than 55–64 year old males.

**Fig 5:**
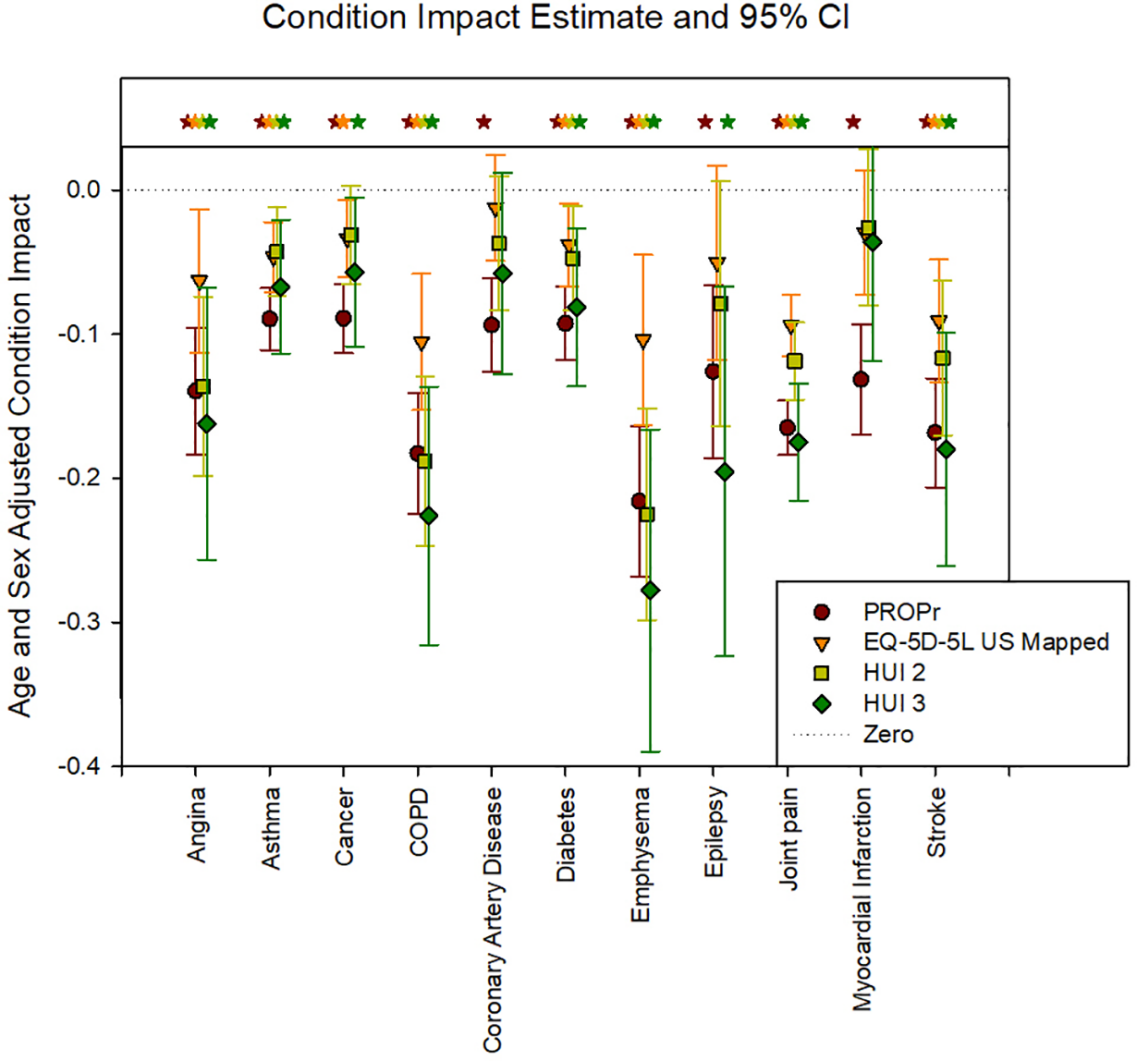
Age- and gender-stratified health condition impacts in the PROPr Estimation sample. HUI 2 is the Health Utilities Index Mark 2, HUI 3 is the Health Utilities Index Mark 3, EQ-5D US Mapped is the Euroqol-5D-5L mapped to the US valuation set, PROPr is the PROMIS-Preference score. Statistically significant estimates are indicated with a star.

**Fig 6:**
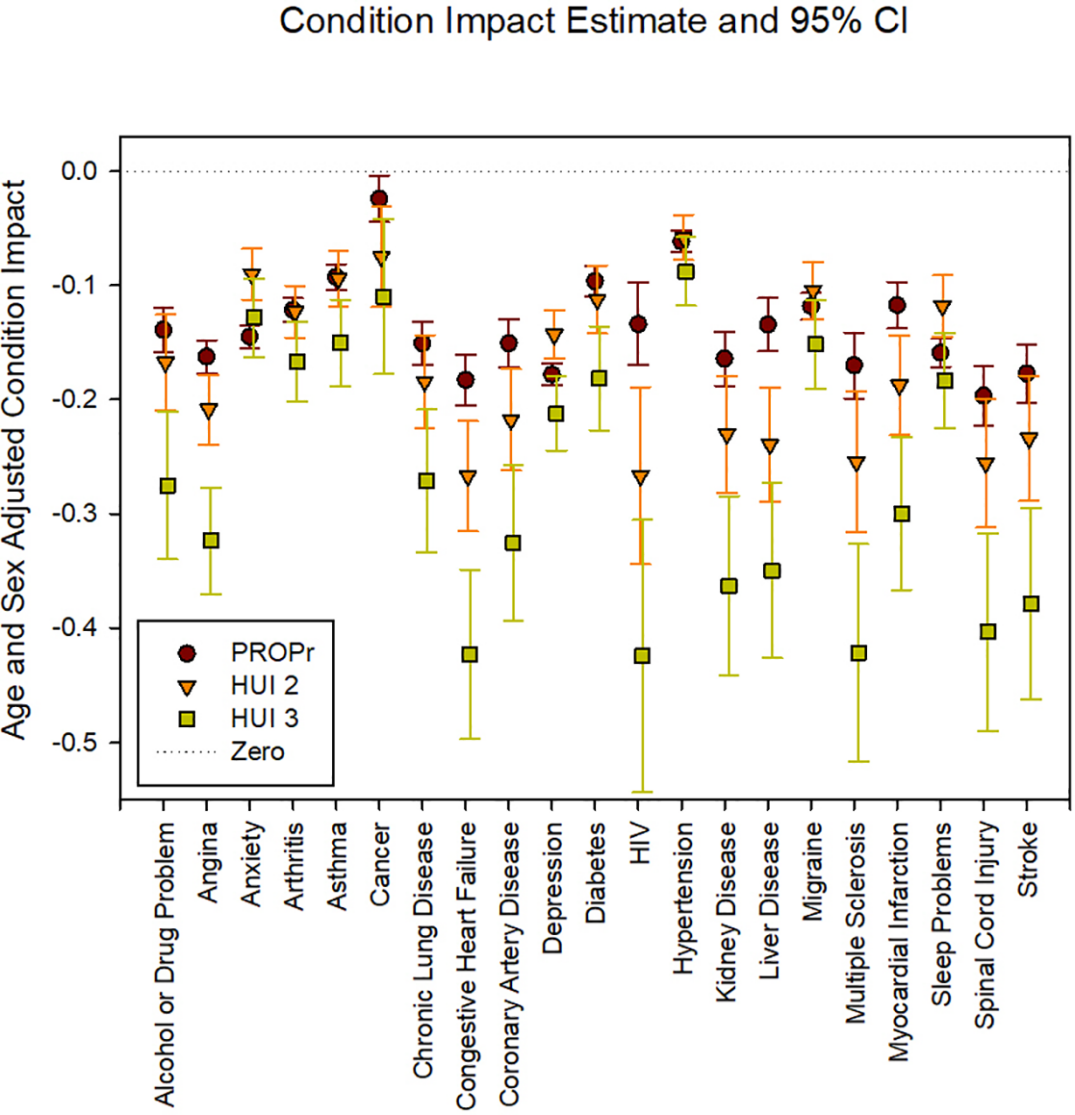
Age- and gender-stratified health condition impacts in the Profiles-HUI sample. HUI 2 is the Health Utilities Index Mark 2, HUI 3 is the Health Utilities Index Mark 3, PROPr is the PROMIS-Preference score. All estimates are statistically significant.

Figs 5 and 6 illustrate the age- and gender- adjusted health condition impacts in the PROPr Estimation and Profiles-HUI samples, respectively. In the PROPr Estimation survey, all 11 condition impact estimates were statistically significantly different from zero (p<0.05) using PROPr, 8 were statistically significant by the EQ-5D, 7 were statistically significant by HUI Mark 2, and 9 were statistically significant by HUI Mark 3. In the Profiles-HUI survey, all 21 condition impact estimates were statistically significant from zero (p<0.05) for all summary scores by all three scoring systems.

The average condition impact as measured by PROPr is most similar to the HUI Mark 3 in the PROPr Estimation survey (average is -0.136 for PROPr, -0.138 for HUI Mark 3, -0.095 for HUI Mark 2, and -0.061 for the EQ-5D) whereas is it most similar to the HUI Mark 2 in the Profiles-HUI survey (average is -0.137 for PROPr, -0.173 for HUI Mark 2, and -0.268 for HUI Mark 3). HUI Mark 3 generally has the largest impact estimates in both surveys. The standard error of these coefficients was smallest for PROPr in both the PROPr estimation survey (average is 0.036 for PROPr, 0.077 for HUI Mark 3, 0.051 for HUI Mark 2, and 0.041 for EQ-5D) and the Profiles-HUI survey (average is 0.021 for PROPr, 0.045 for HUI Mark 2, and 0.069 for HUI Mark 3). The correlation of conditions by impact estimate is similar across all summary scores with a Pearson correlation greater than 0.70 for all comparisons and a Spearman correlation greater than 0.68 for all comparisons.

## Discussion

This report provides the first evaluation of the validity of the PROPr score using 2 large cross-sectional datasets from the general US population. We found that PROPr has good convergent validity with 2 other preference-based summary measures of health–the EQ-5D and the HUI. We also found that PROPr discriminates between those with and without a variety of chronic health conditions. The relative impact of these chronic conditions, as measured by PROPr, was similar to the other preference-based summary scores. Taken together, these findings provide solid justification for the use of PROPr to quantify health-related quality of life for a variety of uses, including calculating aggregated indices of morbidity and mortality such as QALYs.

In these samples, the correlation between PROPr and the other summary measures ranged from 0.66 to 0.70. A prior study that co-administered preference-based summary measures in a large general US population found correlations from 0.60 to 0.71 [31, 33]. The maximum correlation between two measures is the square root of the product of their reliabilities; since the reliability of most health utility measures is below 0.75, the maximum correlation would be below 0.75. Note that the correlations between HUI Mark 2 and HUI Mark 3 are inflated because some of the same questions were used to create both scores. It is also important to note that cross sectional correlations do not necessarily predict correlations in longitudinal change scores [34, 35].

Although PROPr scores correlate well with EQ-5D and HUI scores, PROPr scores are generally lower than corresponding EQ-5D, HUI2, and HUI3 scores. We expected PROPr scores to be lower than these legacy measures because the best possible health state described in PROPr is qualitatively much better than the best health state described in legacy measures. For example, the highest physical functioning level in PROPr is “able to dress yourself, including tying shoelaces and buttoning up your clothes without any difficulty *and* able to run 100 yards (100 m) without any difficulty.” In contrast, the highest physical functioning level in the EQ-5D is “I have no problems walking,” and in the HUI Mark 3, “I have full use of 2 hands and 10 fingers *and* I am able to walk around the neighbourhood without difficulty, and without walking equipment.” The increase in the effective range of measurement of PROPr “raises the bar” to reach a best-health score of 1.0. As a result, ceiling effects in the general population are less common and mean and median scores are substantially lower with PROPr relative to legacy measures. Another explanation of this finding is that PROPr scores analyzed in this report were collected using at least 32 questions, which provides respondents more opportunities to report not-best health status when compared with the other questionnaires (e.g., there are only 5 questions scored for the EQ-5D).

The PROPr scoring system was constructed using the input of experts in IRT-based health profile measurement and preference measurement, as well as the guidance of community members. The final scoring algorithm was estimated using multi-attribute utility theory and was based on preference data from a large representative sample of the US noninstitutionalized population. PROPr is the first score to link single-attribute utility functions to health domains as measured by IRT. As such, PROPr gains the advantages of an IRT-based descriptive system, including flexible administration of items from the 7 item banks used to construct PROPr and finer granularity of its utility scale than those produced by other scoring systems.

The patterns of age- and gender-stratified means in preference-based summary scores for these samples did not match the patterns seen in representatively sampled surveys such as the Medical Expenditures Panel Survey [31, 36]. In particular, males had lower mean scores than females in several age strata and the association between age group and mean score was not monotonic. These differences suggest that the samples used in this report, while drawn from the general US population, are not representative of the general US population. Thus, the reported mean values should not be used as national normative values [37]. Likewise, the health condition impact estimates may not be fully applicable to other studies.

Even though all scores used in this study have different possible score ranges, all are anchored with “dead” anchored at 0 and “full health” anchored at 1.0 and are as a rule not re-scaled for use in cost-utility analyses. PROPr had lower mean scores than the EQ-5D-5, HUI2, or HUI3, but the condition impact measured by PROPr had a similar magnitude to the HUI3 in the PROPr Estimation survey and the HUI2 in the Profiles-HUI survey. That different health utility measures provide different estimates for both cross-sectional differences between groups and longitudinal change within groups has been observed in several other studies [31, 34, 35]. Comparisons of disease impact across surveys, despite collecting some of the same self-reported disease statuses, is limited because disease severity was not measured and there may be differences in disease severity or symptom status. The estimated impact of the chronic conditions was both in the expected direction (the group with a condition has a lower mean than the group without that condition) and statistically significant. Future work will be needed to establish thresholds for a clinically meaningful difference in PROPr scores, as these also differ among preference-based scores.

## Conclusion

The findings presented in this report provide evidence of construct validity for PROPr–it is correlated with other widely used generic preference-based summary scores and those with chronic illness have lower scores than those without the illness. Future work is needed to collect PROPr scores from a nationally representative sample, create crosswalks to legacy measures, and validate PROPr using longitudinal data collection.
