# Correction: Cross-sectional validation of the PROMIS-Preference scoring system

**DOI:** 10.1371/journal.pone.0337044

**Published:** 2025-11-19

**Authors:** Janel Hanmer, Barry Dewitt, Lan Yu, Joel Tsevat, Mark Roberts, Dennis Revicki, Paul A. Pilkonis, Rachel Hess, Ron D. Hays, Baruch Fischhoff, David Feeny, David Condon, David Cella

After publication of this article [1], it was noted that the data in the Dataverse link in the Data Availability statement is deaccessioned. The dataset found previously at https://dataverse.harvard.edu/dataset.xhtml?persistentId=doi:10.7910/DVN/P7UKWR can now be found at https://dataverse.harvard.edu/dataset.xhtml?persistentId=doi:10.7910/DVN/VVNWJX.

The Data Availability statement is updated to: All data files are available from the Open Science Framework (https://osf.io/t896p/) and (Dataverse: https://dataverse.harvard.edu/dataset.xhtml?persistentId=doi:10.7910/DVN/VVNWJX).

In addition, a representative of Northwestern University stated that the OP4G data used in [1] was collected in 2013 by Northwestern and that the data underlying [1] were recently re-reviewed to assess whether there were any anomalies or unexpected results. They stated that no concerns regarding the validity of the data were identified and that Northwestern does not have any concerns regarding this article [1].
